# Complete Genome Sequence of Porcine Circovirus Strain YiY-3-2-H5 with a Novel Insertion, Isolated from Hunan Province, China

**DOI:** 10.1128/genomeA.00151-16

**Published:** 2016-03-31

**Authors:** Qian Gong, Yi Hu, Yang Zhan, Dongliang Wang, Naidong Wang, Guanyu Chen, Aibing Wang, Yi Yang

**Affiliations:** College of Veterinary Medicine, Hunan Agricultural University, Changsha, China

## Abstract

The complete genome of porcine circovirus type 2 (PCV2) strain YiY-3-2-H5 contains a cytidine insertion at position 962 in open reading frame 1. This insertion causes a reading frameshift of the rep gene, and thereafter a premature stop codon is present at the 3′ terminal end of this gene.

## GENOME ANNOUNCEMENT

Porcine circovirus 2(PCV2) is a single-stranded DNA virus, approximately 1.76 kb in genome size and belongs to the genus *Circovirus* within the *Circoviridae* family (1, 2). PCV2 has been identified as the causative agent of porcine circovirus associated disease (PCVAD), a severe disease in the swine industry that causes significant economic loss in many countries (3–5). PCV2 has three major open reading frames (ORFs). ORF1 encodes Rep and Rep′ proteins associated with DNA replication, ORF2 encodes the major structural capsid protein (Cap) (6, 7), and ORF3 encodes a nonstructural protein which is involved in apoptosis in PCV2-infected cells (8). PCV2 is divided into three major genotypes of PCV2a, PCV2b, and PCV2c (9). Currently, PCV2b has been the predominant strain behind the pathogenicity of PCVAD (10).

In this study, a novel PCV2 strain of YiY-3-2-H5 was isolated from various tissue samples (spleen, lymph node, and lung) of a diseased pig with postweaning multisystemic wasting syndrome (PMWS) in the field in Yiyang, Hunan Province, China (11). The PCV2 complete genome was cloned, sequenced, and assembled by DNAStar as previously described (12).

The whole genome of YiY-3-2-H5 strain contains 1,768 nucleotides (nt) and shares 99.89% sequence homology with a known strain of PCV2 SD-QH in GenBank (accession no. KJ511872). Phylogenetic analysis indicates that the YiY-3-2-H5 strain belongs to the cluster of PCV2b-1B. Notably, ORF1 (921 nt) of this strain encodes a carboxyl-terminally truncated Rep protein of 306 amino acids (aa) length. This is 8 aa (EFPYEINY) shorter than the canonical Reps of PCV2 due to a cytidine insertion at position 962 in the coding area of the *rep* gene. ORF2 (*cap* gene) of YiY-3-2-H5 strain shares 100% homology with two known PCV2b isolates deposited in GenBank (accession no. KM880082 and KM360056). The effects of the insertion on the biological function of the Rep and viral DNA replication remain to be determined.

### Nucleotide sequence accession number.

The complete genome sequence of strain YiY-3-2-H5 has been deposited in GenBank under accession no. KU557354.
